# Time and Organizational Cost for Facilitating Implementation of Primary Care Mental Health Integration

**DOI:** 10.1007/s11606-019-05537-y

**Published:** 2019-12-02

**Authors:** Mona J. Ritchie, JoAnn E. Kirchner, James C. Townsend, Jeffery A. Pitcock, Katherine M. Dollar, Chuan-Fen Liu

**Affiliations:** 1 VA Quality Enhancement Research Initiative (QUERI) Program for Team-Based Behavioral Health, U.S. Department of Veterans Affairs, North Little Rock, AR USA; 2 Department of Psychiatry, University of Arkansas for Medical Sciences, Little Rock, AR USA; 3 Central Arkansas Veterans Healthcare System, North Little Rock, AR USA; 4 VA Center for Integrated Healthcare, Syracuse, NY USA; 5 Center of Innovation for Veteran-Centered and Value-Driven Care, VA Puget Sound Health Care System, Seattle, WA USA; 6 Department of Health Services, University of Washington, Seattle, WA USA

**Keywords:** facilitation, implementation research, integrated primary care, mental health, primary care

## Abstract

**Background:**

Integrating mental health services into primary care settings is complex and challenging. Although facilitation strategies have successfully supported implementation of primary care mental health integration and other complex innovations, we know little about the time required or its cost.

**Objective:**

To examine the time and organizational cost of facilitating implementation of primary care mental health integration.

**Design:**

Descriptive analysis.

**Participants:**

One expert external facilitator and two internal regional facilitators who helped healthcare system stakeholders, e.g., leaders, managers, clinicians, and non-clinical staff, implement primary care mental health integration at eight clinics.

**Intervention:**

Implementation facilitation tailored to the needs and resources of the setting and its stakeholders.

**Main Measures:**

We documented facilitators’ and stakeholders’ time and types of activities using a structured spreadsheet collected from facilitators on a weekly basis. We obtained travel costs and salary information. We conducted descriptive analysis of time data and estimated organizational cost.

**Key Results:**

The external facilitator devoted 263 h (0.09 FTE), including travel, across all 8 clinics over 28 months. Internal facilitator time varied across networks (1792 h versus 1169 h), as well as clinics. Stakeholder participation time was similar across networks (1280.6 versus 1363.4 person hours) but the number of stakeholders varied (133 versus 199 stakeholders). The organizational cost of providing implementation facilitation also varied across networks ($263,490 versus $258,127). Stakeholder participation accounted for 35% of the cost of facilitation activities in one network and 47% of the cost in the other.

**Conclusions:**

Although facilitation can improve implementation of primary care mental health integration, it requires substantial organizational investments that may vary by site and implementation effort. Furthermore, the cost of using an external expert to transfer facilitation skills and build capacity for implementation efforts appears to be minimal.

**Electronic supplementary material:**

The online version of this article (10.1007/s11606-019-05537-y) contains supplementary material, which is available to authorized users.

## INTRODUCTION

Integrating mental health services into primary care settings can improve access to mental health care, clinical outcomes, and cost efficiency for patients with mental and behavioral health conditions [1–3]. Such programs are called by various names, e.g., integrated primary care [4], primary care behavioral health [5], integrated care [6], collaborative care management [7], and primary care mental health integration [8, 9]. For this paper, we selected the latter because it is the term utilized in the context in which this study was conducted. Models of primary care mental health integration (PCMHI) are complex and challenging to implement, requiring change in the structure and processes of care delivery [2, 6, 10, 11]; provider values, attitudes, roles, and skills [1, 3, 6, 12, 13]; and organizational culture [6, 14, 15], including ways of working together [6, 12]. Further, primary care settings and their capacity for change vary widely [1, 14–19]. Thus, such programs and the implementation process must be tailored to the needs and resources of the organizational context and PCMHI stakeholders, e.g., leaders, providers, and other staff [10, 17].

Implementation facilitation, a multi-faceted process of interactive problem-solving and support conducted by a designated individual (a facilitator) [20, 21], has successfully supported implementation of complex innovations and care delivery models such as PCMHI [11, 22–26]. To address implementation challenges, facilitators apply a broad range of strategies, including engaging and building relationships with key stakeholders to obtain buy-in and participation, fostering team development, providing education and training, and clarifying stakeholder roles and responsibilities [21, 27–29]. Facilitators also help and enable stakeholders to assess current practices, needs, and resources; plan implementation; monitor implementation progress; conduct ongoing problem identification and resolution; and improve care delivery [20, 21, 26].

Given the complexity of PCMHI programs and potential barriers to implementing them, clinical settings are often unable to implement PCMHI without assistance. Facilitation strategies, particularly those whose costs can be leveraged across more than one practice, are an attractive strategy for providing such assistance. Only two studies, however, have reported facilitation costs and neither addressed PCMHI implementation [30, 31]. Additionally, no studies have accounted for organizational costs associated with stakeholder participation time. This study provides decision-makers with a realistic assessment of the costs of facilitation.

We conducted a large mixed methods project testing a blend of external and internal facilitation within the context of Department of Veterans Affairs (VA) efforts to establish evidence-based PCMHI, including Collaborative Care Management and Co-located Collaborative Care, system-wide [32]. Results showed that clinics receiving facilitation had significantly higher PCMHI reach and adoption, as well as improved program uptake, quality, and adherence to evidence [25, 33]. This article describes the time and effort facilitators and VA stakeholders, i.e., leaders, clinicians, and other staff, devoted to facilitation activities and the organizational cost of facilitation for eight VA primary care clinics. We also sought to understand how facilitators’ time varied across settings, types of activities, and over time.

## METHODS

This descriptive study of facilitation time and cost was conducted from August 2009 through November 2011. The VA Central Institutional Review Board approved and monitored the conduct of the study.

### Study Clinics

To select sites that would receive facilitation, we first selected two VA networks (A and C) based on strength of mental health service line structure, current efforts to support PCMHI implementation, and ability to identify an internal facilitator at 50% effort. Network A had stronger mental health service line structure, operational authority, and existing network level support for PCMHI than network C. The mental health leader in each network identified four primary care clinics that planned to implement PCMHI but would require assistance and served 5000 or more patients. For the evaluation of facilitation’s effectiveness, reported elsewhere, we compared these facilitation clinics to matched clinics in two other networks (B and D) [25, 33]. However, this study focuses exclusively on the eight clinics in networks A and C that received facilitation.

Clinics receiving facilitation varied in size, serving from 4715 to 34,805 primary care patients. Because VA Medical Centers (VAMCs) have administrative responsibility for primary care clinics located at the VAMC and outpatient clinics located elsewhere, facilitation activities with VAMC leaders and managers may influence implementation at any of their clinics. Six of our clinics were under the administration of three VAMCs. For purposes of description and analysis, we grouped the clinics that were under the administration of a common VAMC as paired clinics, including three pairs: A1/A2, A3/A4, and C1/C2. Two clinics (C3 and C4) were under the administration of two different VAMCs; each was measured independently (see Table 1 for additional clinic characteristics).

**Table 1:** Characteristics of Study Clinics

Clinic	Clinic size (no. of PC patients)	No. of PC providers	Location	Academic affiliation	Administration
Network A					
A1 VAMC PC	5632	6	Midwest/urban	Yes	Clinics A1 and A2 under the administration of a common parent VAMC
A2 CBOC	9224	12	Midwest/urban	Yes	
A3 CBOC	4025	6	Midwest/urban	Yes	Clinics A3 and A4 under the administration of a common parent VAMC
A4 CBOC	5654	6	Midwest/urban	No	
Network C					
C1 VAMC PC	34,805	16	Northeast/urban	Yes	Clinics C1 and C2 under the administration of a common parent VAMC
C2 CBOC	14,763	12.6	Northeast/urban	Yes	
C3 CBOC	8125	8	Northeast/urban	No	Clinic C3 under the administration of a different parent VAMC
C4 CBOC	4715	5	Northeast/urban	No	Clinic C4 under the administration of a different parent VAMC

*VAMC* VA Medical Center, *PC* primary care, *CBOC* community-based outpatient clinic

### Implementation Facilitation Strategy

The implementation facilitation strategy consisted of an external facilitator (EF), who worked with and mentored two internal regional facilitators (IRFs), one in each network, to support implementation of evidence-based PCMHI programs. The EF was a national expert in implementation facilitation, PCMHI, and implementation science. The two IRFs, a psychologist and a social worker experienced in PCMHI practices but novice to facilitation, were hired by their network mental health leaders. Although the study supported 50% of IRFs’ and facilitation support staff salaries, our cost estimate accounted for the actual time spent on facilitation activities and did not include time spent on research, e.g., documenting time, or non-study-related activities.

External and internal facilitators helped VA personnel across levels of the healthcare system identify staff who were stakeholders in PCMHI and its implementation. Thus, stakeholders varied by setting. Across settings, facilitators engaged key leaders and managers, PCMHI providers and program managers, primary care providers, nurses, and other staff identified as important. Utilizing a mix of in-person and virtual facilitation, facilitators worked with stakeholders, both individually and in groups, across phases of implementation. Facilitators assessed local contexts and practices; provided academic detailing and education about PCMHI and implementation processes; helped stakeholders design, apply, monitor, and evaluate implementation plans; addressed implementation barriers; and adapted PCMHI to local needs and resources while fostering adherence to evidence-based care delivery models. They also helped train PCMHI providers, established regional learning collaboratives, and helped stakeholders integrate PCMHI into organizational systems and processes. Detailed information about facilitation activities has been previously reported [25, 33] and summarized in Appendix 1.

### Measures and Data Collection

To prepare for documenting facilitation time, MJR/JEK, based on facilitators’ experiences in a pilot study [34], identified types of facilitation activities: assessment, education and marketing, network development, preparation and planning, problem identification and resolution, program adaptation, stakeholder engagement, technical support, IRF training, and travel (see definitions in Appendix 1).

Facilitators documented their activity and travel time on structured spreadsheets (see Appendix 2), collected weekly for the duration of the study, a total of 28 months, generating a total of 1957 records. For each record, facilitators documented time, types of activities, sites that received their services, and the primary activity accounting for most of the time. They excluded research activities. They designated each record (spreadsheet row) as either a “single event,” defined as a meeting or activity of 15 min or more duration, or a “summary” of events, which included multiple activities, each of short duration. Each of the single-event records also listed individual stakeholders who participated in facilitation activities. JCT/MJR reviewed facilitators’ spreadsheets weekly. When records included multiple sites, to avoid overcounting, MJR determined how the time should be allocated by applying a set of rules (see Appendix 3).

To estimate personnel costs, we obtained annual salaries for facilitators and stakeholders through publicly available Federal Salary Web Portals [35, 36]. Salary costs were calculated as the estimated hourly rate multiplied by the time spent on facilitation activities. Cost estimates included the salaries of two network-level facilitation support staff who, according to IRFs, assisted at 25% effort each. Personnel costs included 30% for fringe benefits. We did not account for inflation because the inflation rate was zero during this time period.

Facilitators’ travel expenses included actual travel costs for the EF and estimated IRFs’ costs using General Services Administration mileage and per diem reimbursement rates.

### Data Analysis

We calculated the hours spent by facilitators (EF and IRFs) in support of PCMHI implementation as well as the number of persons (person counts) and hours (person hours) invested by participating stakeholders. We conducted a separate descriptive analysis of travel cost, including salary for travel time and travel expenses.

## RESULTS

### Facilitator and Stakeholder Time Spent on Facilitation Activities

Table 2 summarizes EF and IRF activity and travel time for each network. The EF devoted 141 h in network A and 122 h in network C, approximately 0.05 FTE (excluding travel time), to train IRFs and support implementation at all 8 clinics during the study. The network A IRF (IRF-A) spent more time on facilitation activities (1792 h) than the network C IRF (IRF-C) (1169 h). Travel accounted for a significant proportion of EF time (29% in network A and 50% in network C); including travel, the EF devoted approximately 0.09 FTE to facilitation efforts. Travel only accounted for a small portion of IRF time in both networks.

**Table 2:** Facilitator Activity and Travel Time Across Networks

Facilitators	Network A	Network C
	Hours (%)	Hours (%)
External facilitator (EF)		
Facilitation activities	141 (71)	122 (50)
Travel	57 (29)	121 (50)
EF Total	198 (100)	243 (100)
Internal regional facilitator (IRF)		
Facilitation activities	1792 (91)	1169 (81)
Travel	171 (9)	273 (19)
IRF total	1963 (100)	1442 (100)
Total	2161	1685

Stakeholders across organizational levels participated in facilitation activities (Table 3). In total, 133 and 199 stakeholders participated in networks A and C, respectively. Network A had more network level stakeholders (20 versus 10) and fewer clinic (54 versus 92) and VAMC (46 versus 85) stakeholders than network C.

**Table 3:** Number of Stakeholders and Their Time Spent by Network

Type of stakeholder	Network A		Network C	
	Person counts (%)	Person hours (%)	Person counts (%)	Person hours (%)
Clinic stakeholders	54 (41)	564.5 (44)	92 (46)	571.0 (42)
Key leaders/managers^a^	8	72.0	7	105.5
PCMHI providers^b^	10	343.8	12	269.5
PC providers^c^	13	70.2	22	75.5
PC nurses^d^	6	24.0	10	19.0
MH specialty providers^e^	15	47.5	10	31.5
All others^f^	2	7.0	31	70.0
VAMC stakeholder	46 (35)	352.0 (27)	85 (43)	669.3 (49)
Key leaders^a^	13	112.5	8	168.8
Clinical/operational managers	5	21.5	15	70.8
PCMHI managers/providers^b^	4	112.3	6	133.3
Clinical staff^g^	12	43.2	41	248.0
Non-clinical staff	12	62.5	15	48.4
Network stakeholder	20 (15)	302.1 (24)	10 (5)	69.8 (5)
Key leaders^a^	3	74.7	3	30.5
Program leaders	5	75.5	4	22.5
Non-clinical staff	6	28.5	2	15.8
Consultants	6	123.4	1	1.0
National leaders and other experts	13 (10)	62.0 (5)	12 (6)	53.3 (4)
Total all personnel	133 (100)	1280.6 (100)	199 (100)	1363.4 (100)

*PCMHI* primary care mental health integration, *PC* primary care, *MH* mental health, *VAMC* VA Medical Center

^a^Key leaders and managers including directors/associate directors, chiefs of staff, PC and MH leaders or care line managers, nurse managers, and clinic managers

^b^PCMHI providers included social workers, psychologists, psychiatrists, and nurses who provided mental health services. At the VAMC level, there were also PCMHI program managers

^c^PC providers include MDs, DOs, and nurse practitioners

^d^Nurses include both registered nurses and licensed practical nurses

^e^MH specialty providers include psychiatrists, psychologists, social workers, registered nurses, and addiction therapists

^f^All others at the clinic level included program managers, other professional staff, and support staff who were minimally involved in facilitation activities

^g^PC and MH providers, nurses, and PCMHI providers; in network C, 88% of these were located at VAMCs administratively responsible for but not including study clinics

In total, stakeholders contributed 1280.6 person hours in network A and 1363.4 person hours in network C. In network A, clinic stakeholders devoted the highest number of person hours (564.5), followed by VAMC (352.0) and network (302.0) stakeholders. In network C, clinic and VAMC stakeholders accounted for most of the person hours (571.0 and 669.3, respectively) (see Appendix 4 for more detailed information).

### Organizational Cost of Implementation Facilitation

**Figure 1:**
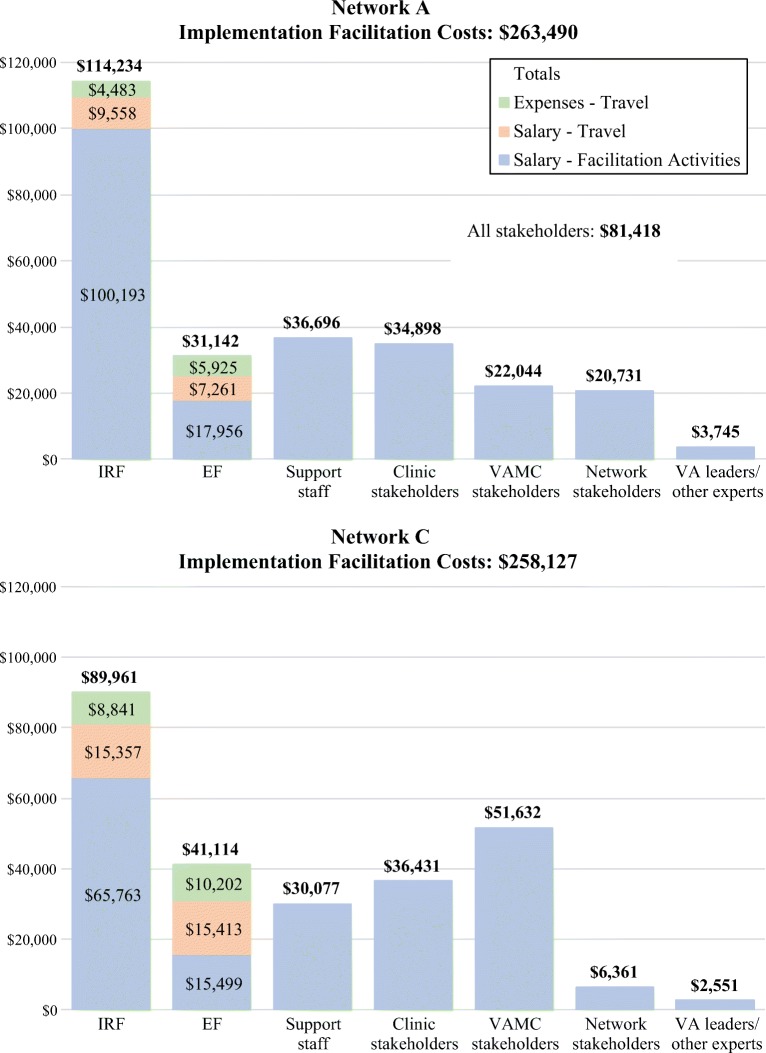
Organizational cost of implementation facilitation. EF external facilitator, IRF internal regional facilitator, VA Department of Veterans Affairs, VAMC VA Medical Center.

The total organizational costs of the implementation facilitation strategy during the 28-month period, including salary support for the EF, IRFs, facilitation support staff, and stakeholders, as well as travel expenses, were $263,490 in network A and $258,127 in network C. Salary support for IRF-A facilitation activities was higher than IRF-C ($100,193 versus $65,763). Network C ($97,975) had a higher stakeholder participation cost than network A ($81,418) (see Fig. 1). The organizational cost of salary support for facililtation activities, excluding travel salary support and expenses, was $236,263 in network A and $208,314 in network C.

### Variation in Facilitators’ Time Across Settings, Types of Activities, and Over Time

The EF and IRFs did not devote equal amounts of time across clinics within each of the networks. For example, in network A, the EF and IRF-A spent relatively more time helping clinic A2 (22%) compared to other clinics. In network C, the EF devoted more time to clinic C2 (19%), while IRF-C devoted more time to clinic C3 (20%). Additionally, IRF-C devoted more time (56%), compared to IRF-A (24%), on activities intended to benefit all clinics in a network (see Table 4 for a detailed comparison of facilitators’ time by network and by clinic).

**Table 4:** Facilitator Time Across Clinics and Regional Networks

Participating clinics	EF	IRF	Total
	Hours (%)	Hours (%)	Hours
Network A			
A1 VAMC PC clinic	16 (11)	318 (18)	334 (17)
A2 CBOC	25 (18)	403 (23)	428 (22)
Both clinics A1 and A2	9 (7)	33 (2)	42 (2)
Total: clinics A1 and A2	50 (36)	754 (42)	805 (42)
A3 CBOC	16 (11)	305 (17)	321 (17)
A4 CBOC	19 (13)	281 (16)	300 (16)
Both clinics A3 and A4	4 (3)	17 (1)	20 (1)
Total: clinics A3 and A4	38 (27)	602 (34)	641 (33)
Across network A	52 (37)	436 (24)	488 (25)
Total—network A	141 (100)	1792 (100)	1933 (100)
Network C			
C1 VAMC PC clinic	16 (13)	64 (5)	80 (6)
C2 CBOC	23 (19)	101 (9)	125 (10)
Both clinics C1 and C2	2 (2)	4 (< 1)	6 (< 1)
Total: clinics C1 and C2	42 (34)	169 (14)	210 (16)
C3 CBOC	12 (10)	230 (20)	241 (19)
C4 CBOC	20 (17)	111 (9)	131 (10)
Across network C	48 (39)	660 (56)	708 (55)
Total—network C	121 (100)	1169 (100)	1290 (100)

Across network A and across network C indicate time spent in facilitation activities with or intended to benefit all of the clinics in network A or network C, e.g., learning collaborative calls for clinics’ PCMHI providers

*EF* external facilitator, *IRF* internal regional facilitator, *VAMC* Department of Veterans Affairs medical center, *PC* primary care, *CBOC* community-based outpatient clinic

**Figure 2:**
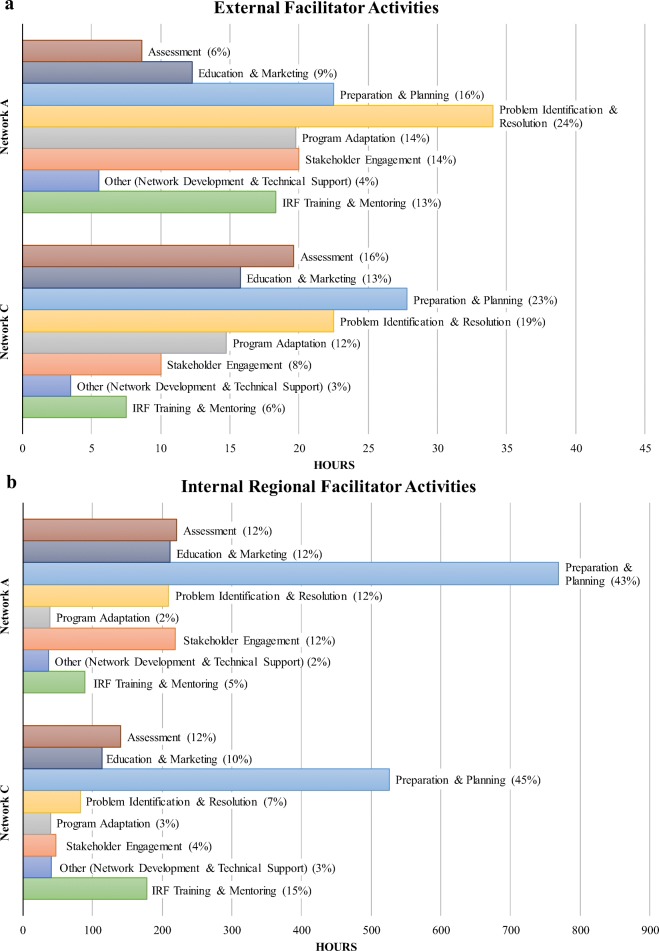
Facilitator hours by primary activity. The charts above illustrate the hours and percentages of time facilitators spent on primary activities in each network. **a** illustrates the external facilitators’ hours and percentages of time on facilitation activities in networks A and C; **b** illustrates the internal regional facilitators’ hours and percentages of time.

The results show variation across facilitators in the proportion of time each spent on particular primary activity categories (Fig. 2). In both networks, the EF spent proportionally more time on problem identification and resolution (24% in network A and 19% in network C) and preparation and planning (16% in network A and 23% in network C) than on other activities. Compared to network C, the EF devoted relatively more time in network A to problem identification and resolution (24% versus 19%), stakeholder engagement (14% versus 8%), and training and mentoring IRF-A (13% versus 6%) and less time on assessment (6% versus 16%) and preparation and planning activities (16% versus 23%).

Both IRFs spent the most time on preparation and planning as a primary activity (43% for IRF-A and 45% for IRF-C), and they were working alone for most of this time. IRF-A spent proportionally more time, compared to IRF-C, on stakeholder engagement (12% versus 4%) and problem identification and resolution (12% versus 7%) and proportionally less time on IRF training and mentoring (5% versus 15%).

**Figure 3:**
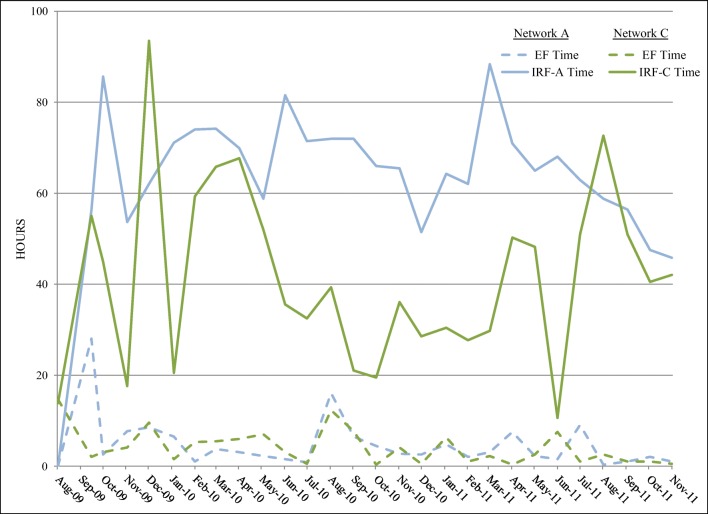
External facilitator and internal regional facilitator time by month. EF external facilitator, IRF internal regional facilitator.

Figure 3 displays the number of hours spent by each facilitator by month over the course of the study. The EF’s time in both networks was relatively stable, tapering off in the last 4 months. Time spent by IRF-A was also relatively stable compared to IRF-C. Both IRFs were continuing to provide support at the end of the study.

## DISCUSSION

This is the first study to assess the time, effort, and organizational cost of facilitating PCMHI implementation. Additionally, it is the first study of facilitation cost to include stakeholders participating in implementation facilitation activities, which is vital for implementation success [37, 38]. Our study found that clinics which needed assistance to implement PCMHI may require substantial organizational investment in resources. We also found that time and cost varied across settings, types of activities, and over time. Study findings provide important insights for planning and applying facilitation strategies.

The substantial amount of resources utilized to facilitate implementation, ranging from $208,314 to $236,263 per network excluding travel cost, is not surprising given the complexity of PCMHI [1, 6, 13], the difficulty of changing the structure and processes of care [15, 39, 40], the large number of contextual barriers that can hinder implementation [10, 12, 15, 16, 19], and the number of stakeholders that might need to be involved. Our study findings support the need to protect time for stakeholder participation in facilitation efforts. Furthermore, significant IRF time was dedicated to implementation throughout the study period, suggesting that some of the clinics needed ongoing assistance for an extended period of time. Based on our data, intensive facilitation strategies like this one can be costly and are likely not required for all sites to successfully implement PCMHI. Future research should explore how to identify sites likely to benefit from facilitation and the dose and duration of facilitation needed to address implementation barriers.

Variation in the type and amount of resources utilized across settings have implications for clinical leaders, managers, and researchers planning to apply facilitation strategies [41]. For example, although our previously reported findings suggested that clinics C1, C2, and C4 had more implementation challenges [33] and therefore might need more assistance, this study shows that IRF-C devoted more time to clinic C3, which was under the administration of the facility where she was located. Perhaps convenience or leadership expectations influenced IRF-C’s use of time. Harvey and colleagues suggest that ongoing monitoring of facilitator time, activities, and fidelity to the facilitation model may increase attention to sites with the highest need and ultimately implementation outcomes [42]. Unfortunately, in this study, the EF could not monitor IRFs’ time. Further exploration of how best to transfer facilitation knowledge and skills from external to internal facilitators as well as tools to monitor fidelity to the implementation facilitation model are needed.

Additionally, IRF-C devoted considerably less time than IRF-A to facilitation efforts, possibly due to competing demands and or differences in career stage, e.g., IRF-C was preparing for retirement and IRF-A was early in her career. On the other hand, IRF-C spent relatively more time on activities intended to benefit all four clinics in the network, e.g., organizing network-wide educational meetings. This was consistent with her previous experiences but was likely also related to contextual factors, e.g., network C lacked infrastructure support for PCMHI implementation present in network A at the start of the study. Our findings suggest that personal factors should be considered when selecting facilitators, and those to whom facilitators are accountable need to protect time for facilitation efforts, ensure that facilitation is provided based on need for assistance, and match facilitation strategies to organizational need as well as facilitator skills.

We encountered several unexpected findings. First, the time and cost of external facilitation, which included mentoring IRFs, were surprisingly minimal. This finding suggests that the facilitation strategy is an efficient model for transferring facilitation skills to develop novice facilitators into experts, thus building implementation capacity for future initiatives. Future research should explore methods and techniques for transferring these skills. Second, although facilitation is a highly interactive process [21, 27, 34], IRFs documented the largest proportion of their time across the length of the study on preparation and planning activities. It is likely that facilitators spent some of this time developing processes and tools that could be applied in subsequent implementation efforts, thus representing an initial time investment that would decrease preparation and planning time in future facilitation efforts. Therefore, there is potential for an “economy of scale” as facilitation is spread to additional sites, which should be explored in future studies.

This study has several limitations. First, we explored time and cost from the perspective of only two inter-related cases (the EF and IRF dyad in two regional networks). Second, we conducted the study in eight clinics in a large US integrated healthcare system for a veteran population. However, the facilitation strategy is applicable to a broad range of healthcare settings within and outside of this country [3]. The final limitation is related to the challenges of collecting time data, including the burden of documentation and the potential for inconsistencies when more than one individual documents time. [43, 44] To reduce burden, the facilitators summarized their time and/or documented multiple activities on some records, which may have resulted in underreporting stakeholder time and facilitation time spent on activities that were not identified as primary activities. Therefore, our time and cost estimates may be lower bound estimates. To ensure data quality, we conducted a rigorous review process and ongoing training. Despite the limitations, our findings provide a baseline for future studies and our study methods are being adapted and applied in other facilitation studies [45].

In summary, the study facilitation strategy, designed to address both the complexity of PCMHI programs and implementation challenges in primary care settings, requires substantial organizational investments that may vary by clinic, healthcare system, stakeholder involvement, and facilitation approach. However, the transfer of facilitation skills from an external expert to initially novice facilitators inside their healthcare systems required minimal resources and built capacity for future implementation efforts. Recognizing its value, VA national mental health leadership adopted this facilitation strategy to support implementation of PCMHI and other initiatives in facilities across VA [32]. Our findings provide useful information to organizations, researchers, and policy makers seeking to implement PCMHI, particularly in clinical settings needing additional implementation assistance.

## Electronic supplementary material

ESM 1 (DOCX 498 kb)
