# Supplementary material for: Time and Organizational Cost for Facilitating Implementation of Primary Care Mental Health Integration
Source: J Gen Intern Med. 2019 Dec 2;35(4):1001–10. doi: 10.1007/s11606-019-05537-y (PMC7174254; doi:10.1007/s11606-019-05537-y)
Supplement: Supplementary file 1 — (DOCX 498 kb) [file 11606_2019_5537_MOESM1_ESM.docx]

**Appendix 1**

**Facilitation Activity Categories: Definitions and Example Activities***

**Note:** Primary Care Mental Health Integration (PCMHI) is the U.S. Department of Veterans Affairs name/acronym for Integrated Primary Care.

**Assessment**

Definition of activity category: Assessment involves gathering information about the clinic and organizational context, stakeholders (local, medical center and regional), patient population, and implementation process, progress, and outcomes.

Examples of facilitation activities in this category:

- Working with clinic staff and medical center and network leaders to identify current practice patterns for managing mental health issues in primary care, including, but not limited to, depression, alcohol and PTSD, as well as behavioral health issues such as adherence, pain management and lifestyle changes
- Gathering and reviewing data from formal and informal data sources
- Using gathered information to understand the organizational context/environment (including leadership support; resources available for PCMHI implementation (e.g., space, staffing, and data capture systems); organizational culture; and readiness to change, etc.)
- Identifying site needs, barriers and facilitators to PCMHI implementation
- Providing program summaries to support leadership decisions regarding PCMHI program sustainability and spread
- Using data capture systems to monitor implementation progress, and implementation and clinical outcomes

**Stakeholder engagement**

Definition of activity category: Stakeholder engagement involves building and maintaining relationships with key stakeholders to create an atmosphere of open communication and obtain stakeholder buy-in, support for and participation in implementation activities.

Examples of facilitation activities in this category:

- Engaging regional and medical center managers directly through presentations about PCMHI programs
- Engaging local management and front-line providers through briefings and individual meetings during clinic visits
- Incorporating process of implementation feedback from the implementation effort into existing leadership meetings and information dissemination mechanisms
- Keeping key stakeholders updated on the progress, obstacles, relevant data, impact on the organization, and implementation successes
- Having routine conversations with key stakeholders through emails and quick phone calls to ensure they remain invested and actively engaged
- Working with stakeholders to overcome resistance to change

**Education and marketing**

Definition of activity category: Education and marketing involves providing information to stakeholders about PCMHI, the evidence for PCMHI, stakeholder roles and responsibilities, PCMHI care delivery processes, implementation processes, and the benefits of implementing PCMHI (for patients and the organization).

Examples of facilitation activities in this category:

- Providing briefings about PCMHI programs and previous outcomes to medical center management to ensure they are aware and supportive of the PCMHI Initiative
- Educating all clinic primary care and mental health personnel on appropriate PCMHI program components, how to access or use them, and how PCMHI programming will affect them
- Training and mentoring PCMHI providers on how to deliver PCMHI services, monitor their work, identify and screen patients, and improve their services
- Providing local change agents with educational tools that can facilitate implementation
- Coordinating regional and clinic level educational efforts
- Providing educational briefings about the PCMHI program to other QI initiatives that organizations are implementing
- Educating PCMHI staff on the interpretation of performance feedback reports
- Supporting marketing activities at clinics
- Adapting a marketing toolkit
- Providing the toolkit to clinics in brief face-to-face meetings with local change agents and providers
- Identifying ongoing marketing opportunities and strategies through PCMHI program meetings

**Program adaptation**

Definition of activity category: Program adaptation involves working with local stakeholders to adapt evidence-based PCMHI care delivery models to clinic needs, resources, and context and helping them maintain fidelity to those models.

Examples of facilitation activities in this category:

- Meeting with primary care, mental health, and nursing leadership, the PCMHI providers, and other key program staff during clinic visits to fit the program to the local context and needs while ensuring fidelity to the program’s core components
- Designing and documenting a local PCMHI implementation plan
- Reviewing adaptations during PCMHI model specific meetings
- Conducting telephone or site visit consultation on adaptation as clinic personnel or program needs change
- Maintaining awareness and contact with other QI initiatives conducted within primary care and mental health to ensure that the PCMHI program is adapted to work synergistically with these initiatives
- Using formative evaluation techniques to support and adapt both the selected PCMHI program and implementation intervention applying the baseline utilization information as well as ongoing performance feedback reports

**Problem identification and resolution**

Definition of activity category: Problem identification and resolution involves working with stakeholders to identify and address barriers or challenges to successful PCMHI implementation.

Examples of facilitation activities in this category:

- Helping sites to identify and address possible barriers to implementation, including problems related to workflow, access to PCMHI services, lack of resources, staff turnover, and role shifting
- Reviewing implementation process outcomes and identifying implementation barriers during monthly program specific implementation teleconferences with integrated personnel
- Assisting sites in problem resolution by communicating with local services and/or senior leadership as appropriate
- Helping sites engage in brain storming, problem-solving and goal setting activities to address challenges
- Identifying and informing sites about best practices used elsewhere to address similar challenges
- Supporting the site in implementing and monitoring trials of local innovations to address challenges

**Network development**

Definition of activity category: Network development involves fostering connections and relationships between local stakeholders or stakeholders across organizations for purposes of sharing information or services.

Examples of facilitation activities in this category:

- Working to create a learning and networking collaborative among clinics implementing similar PCMHI programs during program specific breakout groups in regional education meetings as well as through program-specific teleconferences
- Supporting clinic personnel’s use of the collaborative in conjunction with their own experiences to enable and empower them to address implementation barriers and identify innovative facilitators.
- Encouraging and supporting networking within the collaborative for problem solving and education of new PCMHI providers
- Using the learning collaborative to identify educational needs for PCMHI staff
- Using successful PCMHI providers as educators themselves in regional and local educational efforts and in trainings for other QI initiatives
- Encouraging primary care providers and other stakeholders to share their experiences, including challenges and successes, with similar stakeholders in other settings

**Technical support**

Definition of activity category: Technical support involves providing stakeholders with tools, sample materials, and other resources; helping stakeholders create documents and evaluation materials; working with IT and other stakeholders to ensure accurate data; and providing support for software utilization.

Examples of facilitation activities in this category:

- Working with local Office of Information Technology (OIT) to: develop monthly performance feedback reports which will document the ongoing PCMHI program implementation and process of care outcomes and support the sites’ adherence to performance measures; establish clinics and note templates; and develop monthly/quarterly program reports of diagnosis being seen in the clinics
- Providing materials to assist with hiring appropriate staff (e.g., PCMHI position descriptions, recruitment advertisements, and performance plans)
- Assisting stakeholders with software use and appropriate EMR documentation
- Helping stakeholders design feedback reports and ensure data quality
- Developing or providing stakeholders with educational materials, data collection and tracking forms, or other resources

**Preparation/planning**

Definition of activity category: Preparation and planning involves working alone or collaborating with stakeholders to help sites prepare for implementation or plan activities that will support PCMHI implementation.

Examples of facilitation activities in this category:

- Helping clinics identify local change agents (i.e., champions)
- Helping clinics hire PCMH program staff using developed position descriptions
- Based on assessment activities, facilitating selection of the PCMHI care delivery models that will best meet clinic and regional needs
- Using information and data collected during assessment activities to plan implementation facilitation activities that will best meet local needs and resources
- Assisting stakeholders with setting goals and priorities and developing a shared vision
- Developing presentations for meetings, reviewing data, drafting communications for marketing, preparing implementation meeting agendas, and conducting other general preparation administrative tasks

**Internal regional facilitator (IRF) training**

Definition of activity category: IRF training involves activities targeted to helping the internal facilitators develop facilitations skills.

Examples of activities in this category:

- Attending formal training workshops to increase IRFs’ knowledge of PCMHI models, services or implementation strategies
- Conducting Informal activities (e.g., reading books or articles, reviewing other educational materials, consulting with other PCMHI experts) to increase IRFs’ knowledge of PCMHI care models, services or implementation strategies
- Training/mentoring activities conducted by the expert external facilitator

*The definitions of these activity types have been modified from the initial list provided to facilitators at the beginning of the study. Over time, as we provided ongoing consultation to them on how to document their time, our understanding of what these activities involved expanded. Thus, the definitions in this list include the original definitions but also reflect the activities facilitators documented in each category.

**Appendix 2: Time Tracking Log Sheet**


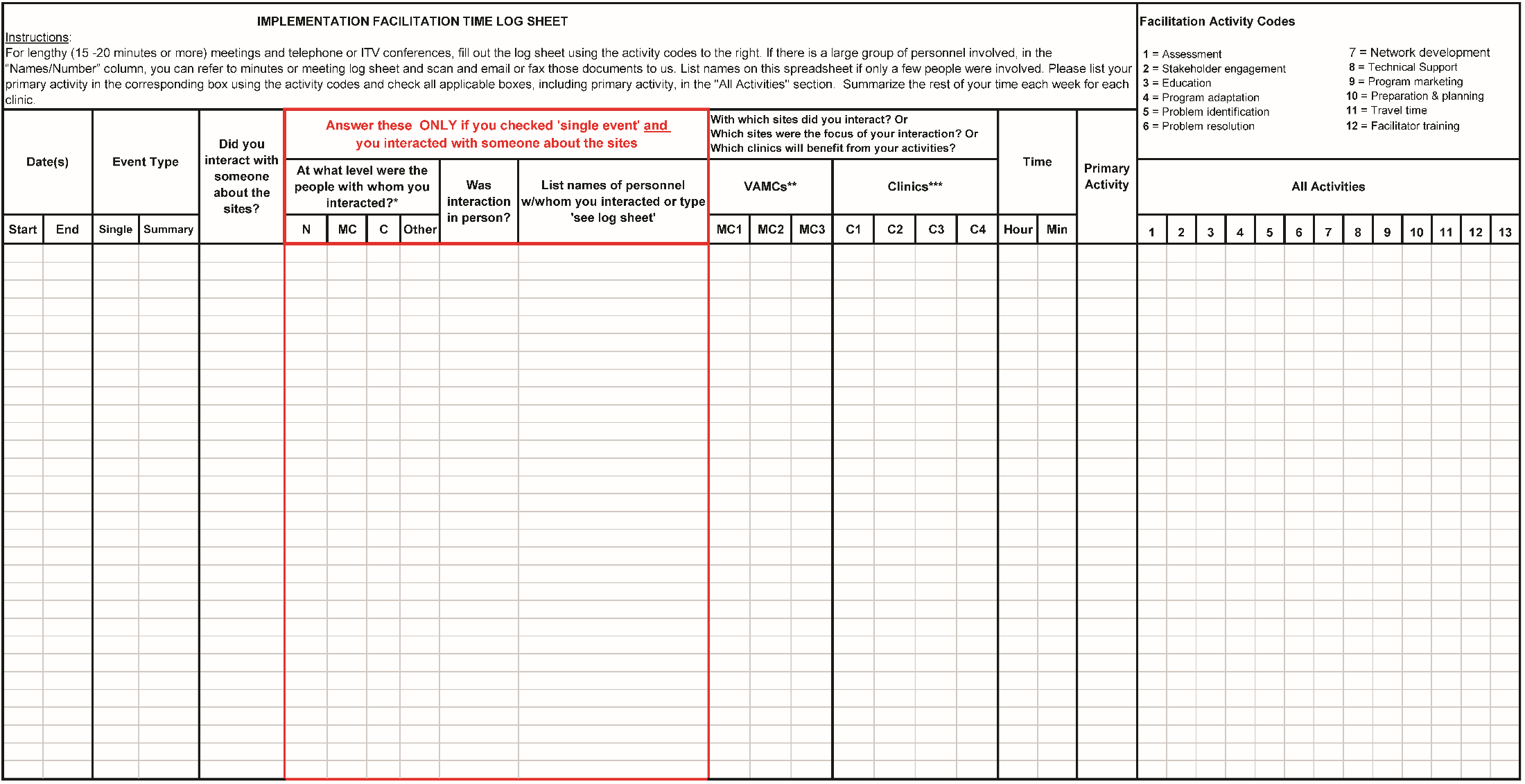


*Levels of interaction (N=Network; MC=Medical center; C=Clinic)

**VAMC=VA medical centers (MC1; MC2; MC3)

***Clinics (C1; C2; C3; C4)

Note: See Appendix 1 for Facilitation Activity Code Definitions

Project in which tracking log was used:

Blended Facilitation to Enhance PCMH Program Implementation (J. Kirchner, PI)

Funded by: VA Quality Enhancement Research Initiative (SDP 08-316)

Data collection: 2009-2011

**Appendix 3**

**Allocation Rules for Records Crediting Multiple Sites**

Facilitators sometimes engaged and worked with stakeholders from multiple clinics and/or VAMCs during the same facilitation event. To avoid overcounting, we identified scenarios that occurred and created rules for allocating time to study clinics for each scenario.

1. Scenario 1: The time record documented a “summary” of events, consisting of multiple activities of short duration, that attributed the time to two or more clinics and/or VAMCs. There was no way to know how much time the facilitator helped each clinic. Rule: Split the time equally between each of the clinics listed on the record.
2. Scenario 2: The time record documented a “single event” in which facilitation activities were targeted to 2 or more clinics individually, e.g., the facilitator was conducting problem-solving for more than one clinic during that block of time. Again, there was no way to know how much time the facilitator helped each clinic. Rule: Split the time equally between each of the clinics listed on the record.
3. Scenario 3: The time record documented a “single event” in which the facilitator was helping both clinics under the administration of a common parent VAMC (e.g., Clinics A1 and A2). Facilitation activities were targeted to the clinics collectively rather than individually. Rule: Allocate all of the time to a category of both clinics, e.g., “Both Clinics A1 and A2.”
4. Scenario 4: The time record documented a “single event” in which facilitation was provided collectively to 3 to 4 clinics, e.g., the facilitator was working with network IT personnel to develop a system for monitoring PCMHI implementation at all 4 clinics or was training or mentoring staff at participating clinics. Rule: Allocate all of the time to a category of all four clinics in the network, collectively, e.g., “Across Network A” (see Table 4).

**Appendix 4**

**Expanded Table 3: Number of Stakeholders and Time Spent**

| **Type of stakeholder** | **Network A** | | **Network C** | |
| --- | --- | --- | --- | --- |
|  | **Person counts (%)** | **Person Hours (%)** | **Person Counts (%)** | **Person Hours (%)** |
| Clinic stakeholders | 54 (41%) | 564.5 (44%) | 92 (46%) | 571.0 (42%) |
| Key leaders/managers | 8 | 72.0 | 7 | 105.5 |
| Primary care mental health integration (PCMHI) providers | | | | |
| Doctors of osteopathy | 1 | 14.0 | 0 | 0.0 |
| Medical doctors | 0 | 0.0 | 3 | 45.0 |
| Nurse practitioners | 1 | 5.0 | 2 | 46.5 |
| Registered nurses | 0 | 0.0 | 1 | 17.0 |
| Social workers | 8 | 324.8 | 6 | 161.0 |
| Primary care providers |  |  |  |  |
| Doctors of osteopathy | 1 | 5.5 | 1 | 8.3 |
| Medical doctors | 8 | 52.7 | 10 | 37.0 |
| Nurse practitioners | 4 | 12.0 | 11 | 30.2 |
| Nurses |  |  |  |  |
| PC registered nurses | 5 | 23.0 | 7 | 12.5 |
| PC licensed practical nurses | 1 | 1.0 | 3 | 6.5 |
| Mental health specialty care providers | | | | |
| Psychiatrists | 5 | 23.5 | 4 | 8.0 |
| Psychologists | 3 | 6.5 | 2 | 7.5 |
| Social workers | 7 | 17.5 | 3 | 13.5 |
| Addiction therapist | 0 | 0 | 1 | 2.5 |
| Clinic program managers | 0 | 0 | 4 | 8.5 |
| Other clinical staff |  |  |  |  |
| Pharmacists/techs | 0 | 0 | 8 | 19 |
| PC social workers | 0 | 0 | 2 | 7.5 |
| Dietician |  |  | 1 | 2.5 |
| Lab staff |  |  | 1 | 1.5 |
| Audiologist | 1 | 2.5 | 0 | 0 |
| Non-clinical staff | 1 | 4.5 | 15 | 31.0 |
| VAMC stakeholder | 46 (35%) | 352.0 (27%) | 85 (43%) | 669.3 (49%) |
| Key leaders | 13 | 112.5 | 8 | 168.8 |
| Clinical/operational managers | 5 | 21.5 | 15 | 70.8 |
| PCMHI managers/providers |  |  |  |  |
| PCMHI managers | 4 | 112.3 | 3 | 86.3 |
| PCMHI providers | 0 | 0 | 3 | 47.0 |
| Clinical staff |  |  |  |  |
| Doctors of osteopathy | 2 | 3.0 | 0 | 0 |
| Medical doctors | 1 | 3.5 | 13 | 64.0 |
| Nurse practitioners | 0 | 0 | 1 | 4.0 |
| Physician assistants | 1 | 4.5 | 0 | 0 |
| Pharmacists | 0 | 0 | 1 | 2.0 |
| Psychologists | 2 | 4.2 | 6 | 25.0 |
| Registered nurses | 0 | 0 | 5 | 34.5 |
| Social workers | 5 | 21.0 | 14 | 116.5 |
| Other | 1 | 7.0 | 1 | 2.0 |
| Non-clinical staff | 12 | 62.5 | 15 | 48.4 |
| Network stakeholder | 20 (15%) | 302.1 (24%) | 10 (5%) | 69.8 (5%) |
| Key leaders | 3 | 74.7 | 3 | 30.5 |
| Program leaders | 5 | 75.5 | 4 | 22.5 |
| Non-clinical staff | 6 | 28.5 | 2 | 15.8 |
| Consultants | 6 | 123.4 | 1 | 1.0 |
| National leaders and other experts | 13 (10%) | 62.0 (5%) | 12 (6%) | 53.3 (4%) |
| Total all stakeholders | 133 (100%) | 1,280.6 (100%) | 199 (100%) | 1,363.4 (100%) |
